# Leonurine-Repressed miR-18a-5p/SOCS5/JAK2/STAT3 Axis Activity Disrupts CML malignancy

**DOI:** 10.3389/fphar.2021.657724

**Published:** 2021-04-16

**Authors:** Hui-Min Liu, Chun-Ling Guo, Yao-Fang Zhang, Jian-Fang Chen, Zhi-Peng Liang, Lin-Hua Yang, Yan-Ping Ma

**Affiliations:** ^1^ Department of Hematology, The Second Hospital of Shanxi Medical University, Taiyuan, China; ^2^ Department of Cardiology, The Second Hospital of Shanxi Medical University, Taiyuan, China; ^3^ Basic Laboratory of Internal Medicine, The Second Hospital of Shanxi Medical University, Taiyuan, China

**Keywords:** leonurine, chronic myeloid leukemia, miR-18a-5p, SOCS5, stat3

## Abstract

Leonurine, an active natural alkaloid compound isolated from Herba leonuri, has been reported to exhibit promising anticancer activity in solid tumors. The aim of this study was to explore whether leonurine is able to inhibit chronic myeloid leukemia (CML) malignancy. Here, we found that leonurine dose dependently inhibited the proliferation, migration, colony formation and promoted apoptosis of CML cells. Furthermore, leonurine markedly reduced CML xenograft growth *in vivo*. Mechanically, leonurine upregulated SOCS5 expression, thus leading JAK2/STAT3 signaling suppression. Silencing of SOCS5 by its siRNA abrogated the effect of leonurine on CML cells, demonstrating that SOCS5 mediates the anti-leukemia effect of leonurine. Notably, we observed that miR-18a-5p was remarkably increased in CML cells. Treating CML cells with leonurine significantly decreased miR-18a-5p expression. Moreover, we found miR-18a-5p repressed SOCS5 by directly targeting its 3′-UTR. miR-18a-5p downregulation induced by leonurine reduced the biological activity of CML cells by relieving miR-18a-5p repression of SOCS5 expression. Taken together, leonurine exerts significant anti-leukemia efficacy in CML by regulating miR-18a-5p/SOCS5/JAK2/STAT3 axis.

## Introduction

Chronic myeloid leukemia (CML) is a clonal myeloproliferative disorder characterized by the Philadelphia chromosome (Nowell and Hungerford, 1961). The fusion of the Abelson gene (Abl) on chromosome nine and breakpoint cluster region (Bcr) on chromosome 22 results in Bcr-Abl oncoprotein expression, which plays a vital role in the pathogenesis of CML by activating multiple mitogenic signaling pathways (Daley et al., 1990; Ren, 2005). Clinical outcome of CML has improved since the introduction of tyrosine kinase inhibitors (TKIs), which are now considered standard therapy for CML patients (Hochhaus et al., 2020a). However, primary and secondary resistance to TKIs hamper the response of anti-leukemia treatment. In addition, many patients also experience TKIs intolerance (Huang et al., 2016; Hochhaus et al., 2020b). Therefore, identifying more effective agents to improve therapeutic intervention for CML patients and better understanding the potential molecular mechanisms of this disease are crucial.

Leonurine is a natural alkaloid extracted from Herba leonuri. A wealth of studies has verified that leonurine can regulate various physiological and pathological processes such as lipid and glucose metabolism, oxidative stress, inflammation, fibrosis and apoptosis (Zhu et al., 2018). For the effect of leonurine on malignant tumors, leonurine was found to inhibit proliferation and induce apoptosis of lung cancer cells through inhibiting mitochondrial pathway (Mao et al., 2015). Leonurine significantly promoted human cervical cancer cell apoptosis and inhibited the expression of P-Gp and MRP1 protein, thus having synergistic antitumorigenic effect with cisplatin (Lin et al., 2020). Sitarek et al. illustrated that the extract of Leonurus sibiricus transgenic roots could induce apoptosis in various grades of human glioma cells by increasing oxidative stress (Sitarek et al., 2017). However, whether leonurine has therapeutic effects on CML is currently elusive.

Accumulating evidences have indicated that microRNAs (miRNAs) have important regulatory effects on oncogenesis and tumor progression, therefore being used as biomarkers for malignant tumor diagnosis and prognosis (Calin and Croce, 2006). miR‐18a‐5p is an important member of the miR-17–92 cluster and was found upregulated in imatinib-resistant compared to imatinib-responsive CML patients (Jurkovicova et al., 2015). However, the precise role and potential molecular mechanism of miR‐18a-5p in CML remain to be elucidated.

JAK2/STAT3 signaling is constitutively hyperactivated in CML and plays an important role in the biology and evolution of CML (Zhao et al., 2020). Hyperactivation of JAK2/STAT3 signaling transduces oncogenic signals to promote CML cells growth, leukemia stem cells survival and therapeutic resistance, thus disease evolution (Stella et al., 2013). JAK2/STAT3 signaling is negatively regulated by many regulators, including the suppressor of cytokine signaling (SOCS) family (Johnson et al., 2018). A recent study suggests that SOCS5 downregulation potentiates aberrant JAK2-STAT3 signal transduction to promote T-cell acute lymphoblastic leukemia (T-ALL) progression (Sharma et al., 2019). However, little is known about the mechanism by which SOCS5 regulates signal transduction in CML.

In the current study, we explored the mechanism of leonurine-mediated anti-leukemia activity in CML. We identified leonurine could suppress CML both *in vitro* and *in vivo*, supporting leonurine as a chemotherapy candidate for CML. The anti-leukemia activity of leonurine was illustrated by regulating miR-18a-5p/SOCS5/JAK2/STAT3 axis.

## Materials and Methods

### Cell and Culture

Human CML (K562 and KU812) and acute myeloid leukemia (AML) cell line (THP-1 and HL60) were purchased from the Shanghai Institute of Cell Biology, Chinese Academy of Sciences (Shanghai, China). The cells were cultured in RPMI-1640 containing 10% fetal bovine serum (FBS; Gibco, Carlsbad, CA, United States) at 37°C with 5% v/v CO_2_. The medium was changed every 3 days.

### Patients and Normal Controls

Peripheral blood mononuclear cells (PBMCs) were collected from 30 newly diagnosed Ph + CML patients and 30 healthy donors at the Department of Hematology of the Second Hospital of Shanxi Medical University from 2018 to 2020. Characteristics of patients and donors are summarized in Table 1. All participants signed the informed consent. The study was approved by the Ethics Committee of Shanxi Medical University.

**TABLE 1:** Patient characteristics.

Characteristics	CML (*n* = 30)	Healthy donors (*n* = 30)
Age (years), median (range)	40.5 (27–65)	31.5 (20–42)
Male/female (n/n)	14/16	21/9
WBC, ×10^9^/L, median (range)	288 (74–478)	6.6 (4.8–10.2)
Hb (g/L), median (range)	104 (89–142)	139.5 (129–158)
PLT, ×10^9^/L, median (range)	284.5 (95–538)	173.5 (122–245)

Hb, hemoglobin; PLT, platelet; WBC, white blood cell.

### Cell Transfection

The miR-18a-5p mimic, inhibitor, si-SOCS5-1#, si-SOCS5-2#, and their respective negative controls were purchased from Sangon Biotech (Shanghai, China). K562 and KU812 cells were plated in 6-well plates at a density of 5 × 10^5^ cells/well and incubated for 24 h. The miR-18a-5p mimics, inhibitor, si-SOCS5-1#, si-SOCS5-2# were transfected into the cells by Lipofectamine 2000 (Invitrogen, Carlsbad, CA, United States) in accordance with the manufacturer’s instructions. At 48 h after transfection, cells were collected for further investigation. Recombinant lentiviruses overexpressing SOCS5 and their respective control lentivirus were obtained from GeneChem (Shanghai, China). For lentivirus infection, cells were cultured in 24-well plates at a density of 5 × 10^4^ cells/well and incubated for 24 h. Then cells were infected the lentiviral vectors at a multiplicity of infection of 50 for 24 h. The medium was replaced with fresh complete medium followed by puromycin (Solarbio Science, Beijing, China) selection of the infected cells. Then cells were collected for gene expression assay and further investigation.

### Real-Time Quantitative PCR

Total RNA was extracted with Trizol (Invitrogen, Carlsbad, CA, United States) according to the manufacturer’s instruction. Reverse transcription was performed with Prime-Script RT reagent Kit (Takara, Dalian, China) or Mir-X miRNA First-Strand Synthesis Kit (Takara, Dalian, China). SYBR Green qPCR Master Mix (Takara, Dalian, China) was used for real-time PCR analysis. U6 and GAPDH were used as the internal controls for microRNA and mRNA, respectively. The comparative ΔΔCT method was used to calculate relative expression. The primers used were as follows: SOCS5: Forward, 5′- GTA​GGA​AGT​CGC​TCT​CTA​AGA​C -3′, Reverse, 5′- TTT​GAC​TGC​TTG​CTG-TAA​GTT​C -3′; miR‐18a‐5p: Forward, 5′‐CGC​TAA​GGT​GCA​TCT​AGT​GCA​GAT​AG‐3′; GAPDH: Forward, 5′‐GAG​TCA​ACG​GAT​TTG​GTC​GT‐3′, Reverse: 5′‐TTG​AGG​TCA​ATG​AAG​GGG​TC‐3′; U6: Forward: 5′‐GCG​CGT​CGT​GAA​GCG​TTC‐3′, Reverse: 5′‐GTG​CAG​GGT​CCG​AGG​T‐3′.

### Cell Viability Assay

Cell viability was evaluated using Cell Counting Kit-8 (Dojindo, Kumamo, Japan) following the manufacturer’s protocols. Leonurine and imainib were bought from Topscience (Shanghai, China). Briefly, cells were seeded into 96-well plates at a density of 3,000 cells/well and treated with leonurine at different concentrations (0, 0.05, 0.1, 0.2, 0.4, 0.8, 1.4, 2.0 mM) for 24 h, or for different times (12, 24, 36, 48, 60 h) at 0.4 mM. To explore whether the effect of imatinib can be enhanced by leonurine, cells were treated with imatinib alone (0, 0.25, 0.5, 1.0, 1.5, 2 µM) or together with leonurine (0.4 mM) for 24 h. Then 10 ul CCK-8 was added to each well and incubated for 2 h at 37°C. The optical density was measured at an absorbance of 450 nm using the SpectraMax ID3 microplate reader (Molecular Devices).

### Cell Apoptosis Assay

Cells were seeded in 6-cell plates at a density of 5×10^5^ cells/well and cultured overnight. Then cells were treated with leonurine, imatinib or both at indicated concentrations for 24 h. Cells were harvested and washed twice with ice-cold PBS. The Annexin V–FITC/7-AAD apoptosis detection kit (BD Biosciences, San Jose, CA, United States) was used to detect cell apoptosis according to the manufacturer’s instructions. The percentage of apoptotic cells were analyzed using the Gallios flow cytometry (Beckman Coulter).

### Western Blotting

Equal amounts of protein were separated by SDS-PAGE, and transferred to a polyvinylidene fluoride membranes (Merck Millipore). The Membranes were blocked with 5% milk at 37°C for 1 h and incubated with primary antibodies as follows at 4°C overnight: SOCS5 (1:1,000, ab97283), JAK2 (1:1,000, CST3230), STAT3 (1:1,000, CST30835), p-STAT3 (1:1,000, phospho Tyr705, CST9145), *β*-actin (1:1,000, CST4970). Subsequently, the membranes were incubated with specific secondary antibodies (1:5,000, CST7074) at 37°C for 1 h. After washing with TBS-Tween (10 min, three times), membranes underwent detection by ChemiDoc XRS + Imaging System (Bio-Rad).

### Soft Agar Colony Formation Assay

Cells were treated with leonurine (0, 0.4, 0.6 mM) for 2 h. Subsequently, Soft-agar assays were added into 6-well plates, each well containing a bottom layer (2 ml) of low-melting 1.2% agarose (Solarbio Science, Beijing, China) and a top layer (2 ml) of 0.7% agarose containing 5,000 cells. Two weeks later, Colonies of more than 50 cells were counted by microscope (Olympus, Tokyo, Japan). The colony formation rate was described by colony number/inoculated cell number ×100%.

### Transwell Migration Assay

The migration assay was performed using a 24-well Boyden transwell chamber (Corning, Cambridge, MA, United States) with 8 μm pore membrane filters. After pretreating with leonurine (0, 0.4, 0.6 mM) for 4 h or transfecting as indicated, cells (1 × 10^4^) were seeded into serum-free RPMI-1640 in the upper chamber. RPMI-1640 supplemented with 10% FBS was added to the lower chamber as the chemoattractant. Cells were cultured for 24 h at 37°C with 5% v/v CO_2_. Then, cells remaining on the upper side of the membrane were removed using cotton swabs. Cells that had migrated through the membranes were fixed in 4% paraformaldehyde for 20 min at 37°C and stained with crystal violet. The cell number was determined in five random fields with a microscope (Olympus, Tokyo, Japan).

### Luciferase Reporter Assay

The wild or mutated 3′ untranslated region (3′-UTR) of SOCS5 were amplified and cloned into firefly luciferase vectors (GenePharma, Shanghai, China) to generate the 3′-UTR wild-type or 3′-UTR mutated reporter plasmid, respectively. K562 cells were transfected with miR-18a-5p mimic or inhibitor, in combination with SOCS5 3′UTR WT or Mut reporter using Lipofectamine 2000. After 48 h, the luciferase activity was detected using luciferase assay kit (Promega, WI, United States) following the manufacturer’s protocol.

### Xenograft Animal Model

Four-week-old female BALB/c nude mice were obtained from Experimental Animal Center of Shanxi Provincial People’s Hospital. Nude mice were implanted subcutaneously with 1x10^7^ LV-miR-inhibitor- or LV-miR-NC-infected K562 cells. All mice formed tumors one week after injection. Mice were assigned randomly into leonurine-treatment and control groups. In the treatment group, from the eighth day, leonurine (150 mg/kg d) was administered to mice via gavage for four consecutive weeks. And the control group was administered with saline. The range of doses of leonurine was selected according to our preliminary experiment indicated that doses higher than 150 mg/kg did not further reduce tumor growth in nude mice. The tumor volume was assessed every three days and calculated using the formula: volume = length × width^2^/2. At the end of the experiment (on day 36), mice were sacrificed and the subcutaneous tumors were excised for H&E staining and immunohistochemistry. This study conformed to the Guidelines on the Care and Use of Laboratory Animals (NIH Publication no.85–23) and was approved by the Animal Care Committee of the Shanxi Medical University.

### Histological and Immunohistochemical Analysis

Tumors removed from mice were fixed with formalin and embedded in paraffin. Four-micrometer thick sections were cut and stained with hematoxylin and eosin (H&E) for histological analysis. Immunohistochemical staining of SOCS5 (1:100, ab97283) was performed following the manufacturer’s instructions. Images were obtained using a microscope (Olympus, Tokyo, Japan).

### Target Prediction

TargetScan (www.targetscan.org), miRDB (mirdb.org), miRanda (www.microrna.org), miRmap (mirmap.ezlab.org) and PITA (https://genie.weizmann.ac.il/pubs/mir07/mir07_prediction.html) were used to identify the potential target microRNA of SOCS5. A Venn diagram was drawn to illustrate the intersection among the five prediction algorithms (http://bioinformatics.psb.ugent.be/webtools/Venn/).

### Datasets for Informatic Analyses of SOCS5 in CML

For assessment of mRNA expression of SOCS5, the microarray data of GSE12211 (Bruennert et al., 2009) were selected from the GEO database (http://www.ncbi.nlm.nih.gov/geo) to analyze (Edgar et al., 2002).

### Statistical Analysis

Each experiment was repeated at least three times. The data are reported as the mean ± standard deviation (SD), and results were analyzed using SPSS26.0 software. All of our data have been tested for normality. The Student *t* test was used to detect the significance of differences between two groups with compliance of homogeneity of variances (Levene’s test), and the one-way analysis of variance (ANOVA) with the post-hoc Dunnett’s test was employed for multiple comparisons with compliance of homogeneity of variances (Levene’s test). Differences were considered significant when *p* < 0.05.

## Results

### Leonurine Inhibits Proliferation, Migration and Induces Apoptosis of CML Cell Lines

**FIGURE 1:**
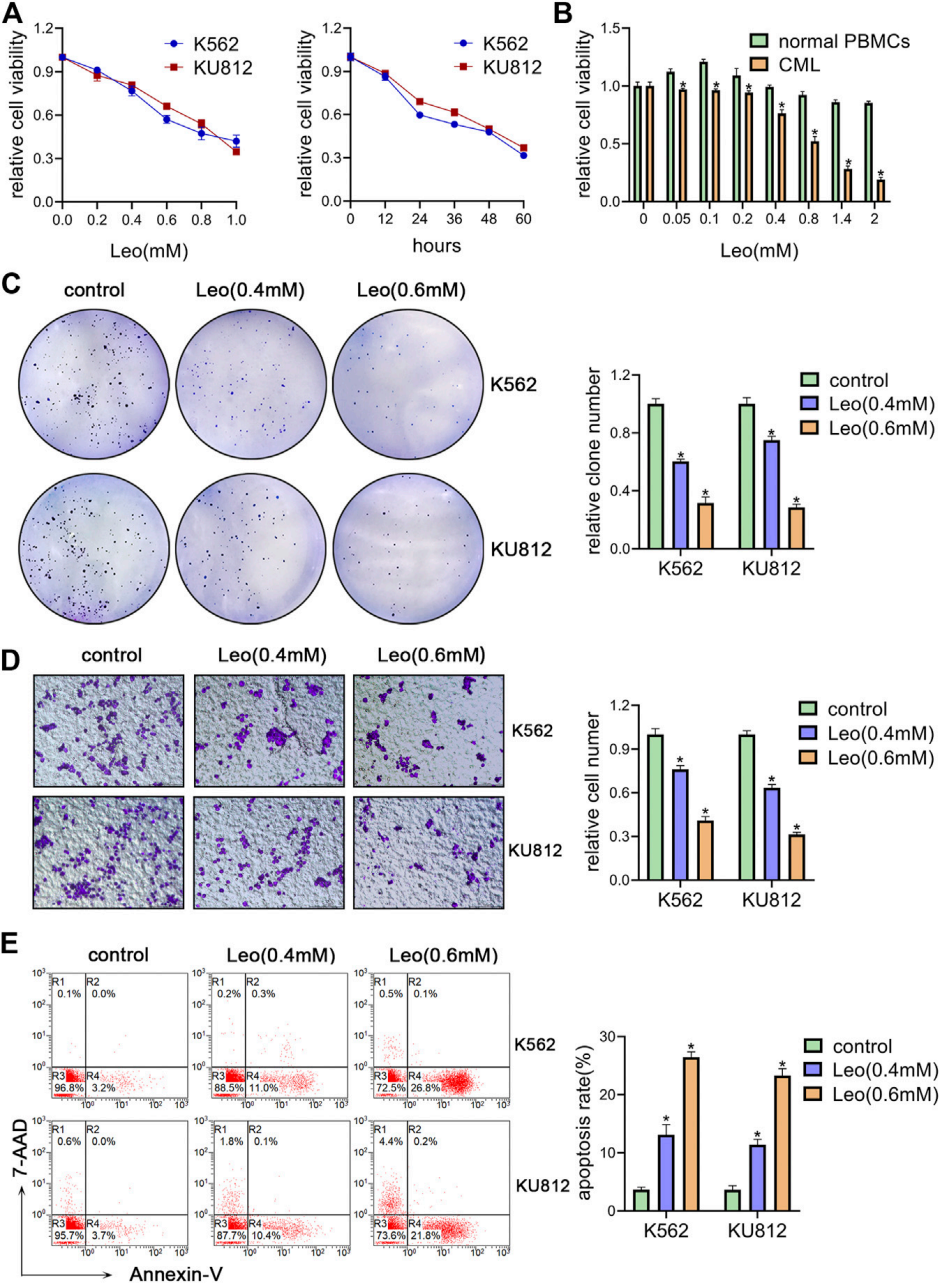
Leonurine inhibits CML cells proliferation, migration and induces apoptosis **(A)** K562 and KU812 cells were treated with leonurine at different concentrations or for different durations. Cell viability was detected by CCK-8 assay **(B)** PBMCs from healthy donors and CML patients were treated with leonurine at different concentrations for 24 h. Cell viability was detected by CCK-8 assay **(C–E)** K562 and KU812 cells were treated with leonurine (0.4 mM or 0.6 mM) for 24 h **(C)** colony formation ability was analyzed by soft agar colony formation assay **(D)** Cell migration was analyzed by transwell assay. Scale bar = 100 μm **(E)** Cell apoptosis was assessed by Annexin V–FITC/7-AAD staining. Results were presented as mean ± SD, and the error bars represent the SD of three independent experiments (n = 3). *p < 0.05 vs. control group.

We first investigated the effect of leonurine on the viability of K562 and KU812 cells. The CCK-8 assay results showed that leonurine inhibited K562 and KU812 cell viability in dose and time dependent manners (Figure 1A). The IC50 of leonurine was 0.773 mM in K562 cells and 0.882 mM in KU812 cells for 24 h treatment. Our data also indicated that leonurine barely affects normal PBMCs (Figure 1B). In addition, leonurine significantly reduced the colony formation capacity of both K562 and KU812 cells compared with the control (Figure 1C). Transwell assay revealed that leonurine suppressed migration (Figure 1D). Moreover, flow cytometry assays revealed that leonurine dramatically induced apoptosis compared with control (Figure 1E). Interestingly, we observed that leonurine mainly induced early apoptosis of CML cells. We also explored whether leonurine could enhance the sensitivity of CML cells to imatinib. K562 and KU812 cells were treated with imatinib of different concentrations together with leonurine for 24 h. The results showed that combined with leonurine dramatically reduced K562 and KU812 cells viability in an imatinib dose-dependent manner compared with imatinib alone (Supplementary Figure S1A). Correspondingly, the combined treatment remarkably increased apoptosis of CML cells compared with imatinib treatment alone (Supplementary Figure S1B). These results indicated that leonurine significantly inhibited cell growth and induced cell apoptosis of CML cell lines.

### Leonurine Suppresses JAK2/STAT3 by Upregulating SOCS5 Expression

**FIGURE 2:**
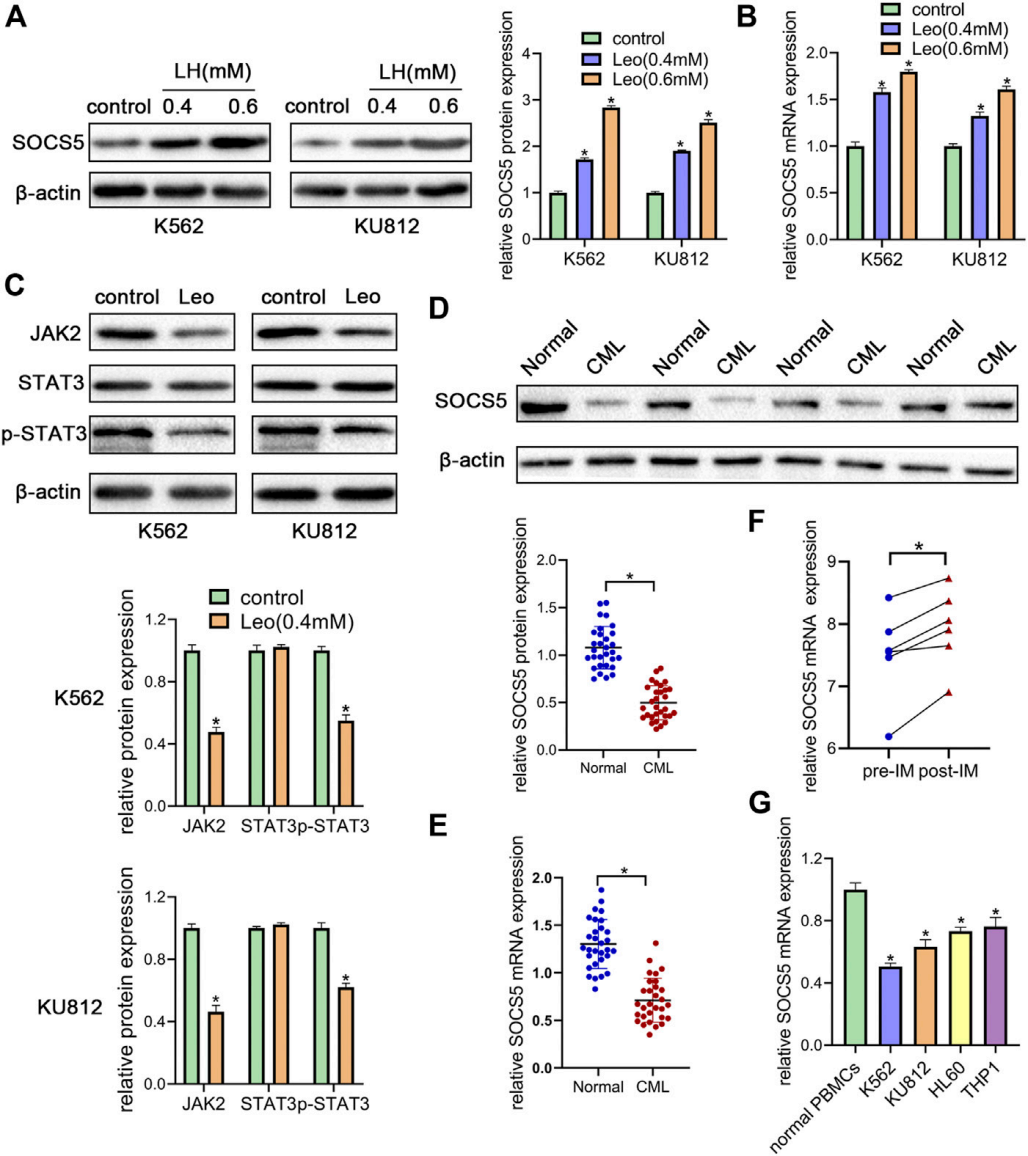
Leonurine suppresses JAK2/STAT3 by upregulating SOCS5 expression **(A)** K562 and KU812 cells were treated with leonurine (0.4 and 0.6 mM) for 24 h. Western blotting was used to detect SOCS5 protein level **(B)** qRT-PCR was used to detect mRNA level of SOCS5 **(C)** K562 and KU812 cells were treated with leonurine (0.4 mM) for 24 h and JAK2/STAT3 signaling protein expression was detected by western blotting **(D)** Western blotting was used to detect SOCS5 protein level in PBMCs **(E)** qRT-PCR was performed to assess SOCS5 mRNA expression in PBMCs **(F)** SOCS5 mRNA expression data before and after imatinib treatment was extracted from GSE12211 **(G)** qRT-PCR was used to detect mRNA level of SOCS5 in leukemia cell lines and healthy PBMCs. Results were presented as mean ± SD, and the error bars represent the SD of three independent experiments (*n* = 3). *p < 0.05 vs. control.

JAK2/STAT3 signaling activation is involved in cellular biological process of proliferation and apoptosis. SOCS5 is a critical negative regulator of JAK2/STAT3 pathway. Hence, we explored the effect of leonurine on JAK2/STAT3 activity and SOCS5 expression. Our results showed that leonurine significantly upregulated SOCS5 expression in a dose-dependent manner in both mRNA and protein level (Figures 2A,B). Conversely, leonurine downregulated JAK2 and phosphorylated STAT3 protein level, while the total STAT3 expression showed no significant change (Figure 2C). These results implied that inhibition of JAK2/STAT3 signaling was associated with SOCS5 upregulation by leonurine.

To uncover the clinical significance of SOCS5 expression, we examined SOCS5 expression of protein and mRNA in newly diagnosed CML patients (*n* = 30). Western blotting and qRT-PCR assay showed lower expression of SOCS5 in CML patients compared with healthy donors (Figures 2D,E). Then we screened publicly available datasets and determined the effect of imatinib treatment on SOCS5 expression. The result indicated that SOCS5 level of CML patients was significantly increased after imatinib treatment (Figure 2F). Furthermore, we detected SOCS5 expression in CML cell lines (K562 and KU812) and AML cell lines (HL-60 and THP-1). SOCS5 mRNA expression was significantly decreased in CML and AML cell lines compared with that in normal PBMCs. Of note, the expression of SOCS5 was lowest in CML cells (Figure 2G). These data revealed that SOCS5 downregulation may contribute to CML development.

### SOCS5 Plays an Essential Role in the Biological Impact of Leonurine on CML Cells

**FIGURE 3:**
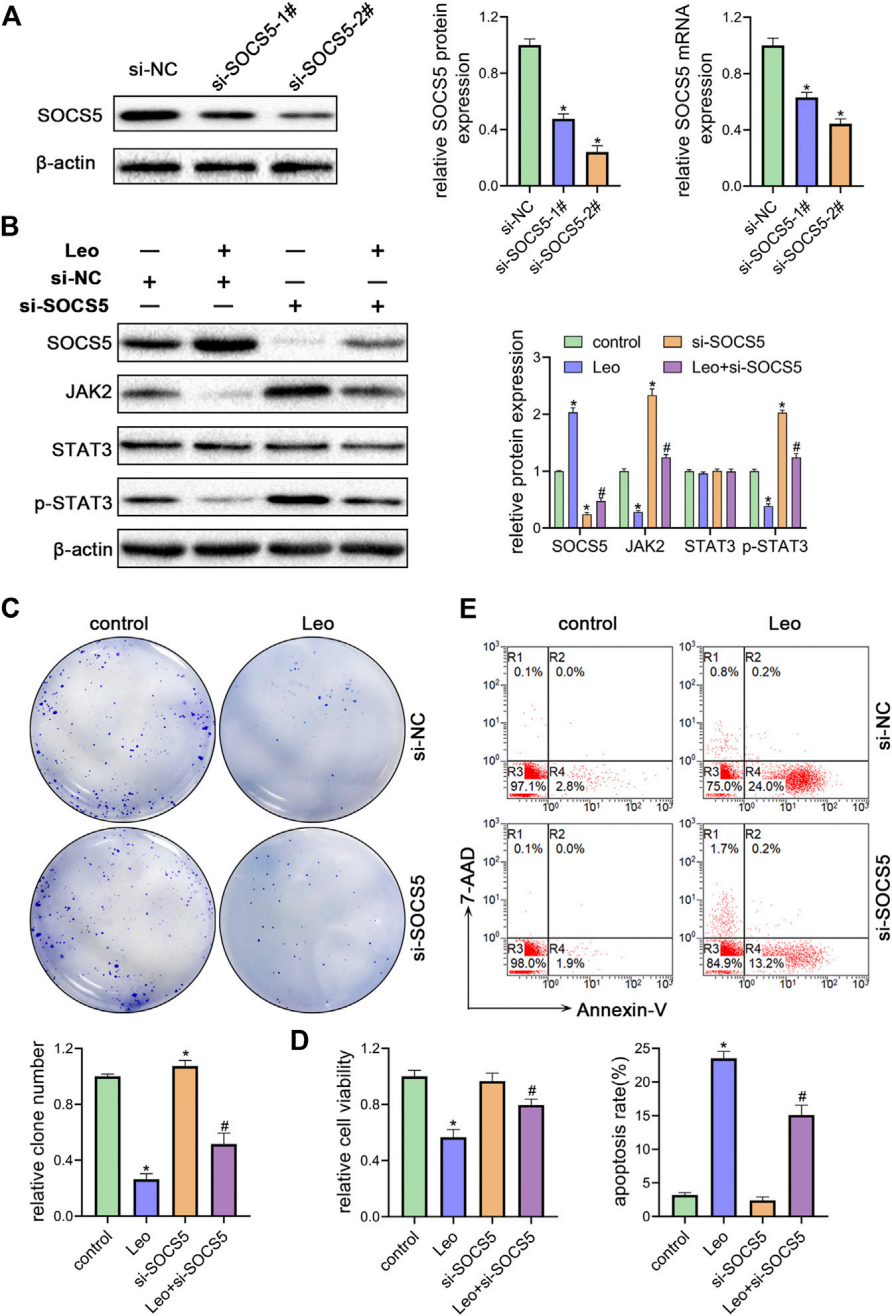
SOCS5 plays an essential role in the biological impact of leonurine on CML cells **(A)** K562 cells were transfected with si-SOCS5-1#, si-SOCS5-2# or si-NC. qRT-PCR and western blotting were performed to detect SOCS5 expression **(B–E)** K562 cells were transfected with si-SOCS5 or si-NC and treated with or without leonurine (0.4 mM) for 24 h **(B)** Protein expressions of SOCS5 and JAK2/STAT3 were examined by western blotting **(C)** Colony formation ability was analyzed by soft agar colony formation assay **(D)** Cell viability was detected by CCK-8 **(E)** Cell apoptosis was assessed by Annexin V–FITC/7-AAD staining. Results were presented as mean ± SD, and the error bars represent the SD of three independent experiments (*n* = 3); *p < 0.05. vs. control group; #*p* < 0.05 vs. Leo group.

Next, two siRNAs were synthesized against SOCS5 (si-SOCS5-1# and si-SOCS5-2#) and were transfected to K562 cells. We confirmed that both siRNAs successfully suppressed SOCS5 expression and si-SOCS5-2# exhibited higher efficiency than si-SOCS5-1# (Figure 3A). Therefore, si-SOCS5-2# was employed to subsequent experiments. We found that knockdown of SOCS5 upregulated JAK2/STAT3 expression (Figure 3B), and thus attenuated leonurine-induced colony reduction (Figure 3C), viability decrease (Figure 3D) and apoptosis elevation (Figure 3E). We also performed the experiments in KU812 cell line and obtained similar results (Supplementary Figure S2). These results indicated that SOCS5 mediated the anti-leukemia process of leonurine.

### miR-18a-5p Upregulation in CML Cells Represses SOCS5 Expression Through Directly Targeting its 3′-UTR

**FIGURE 4:**
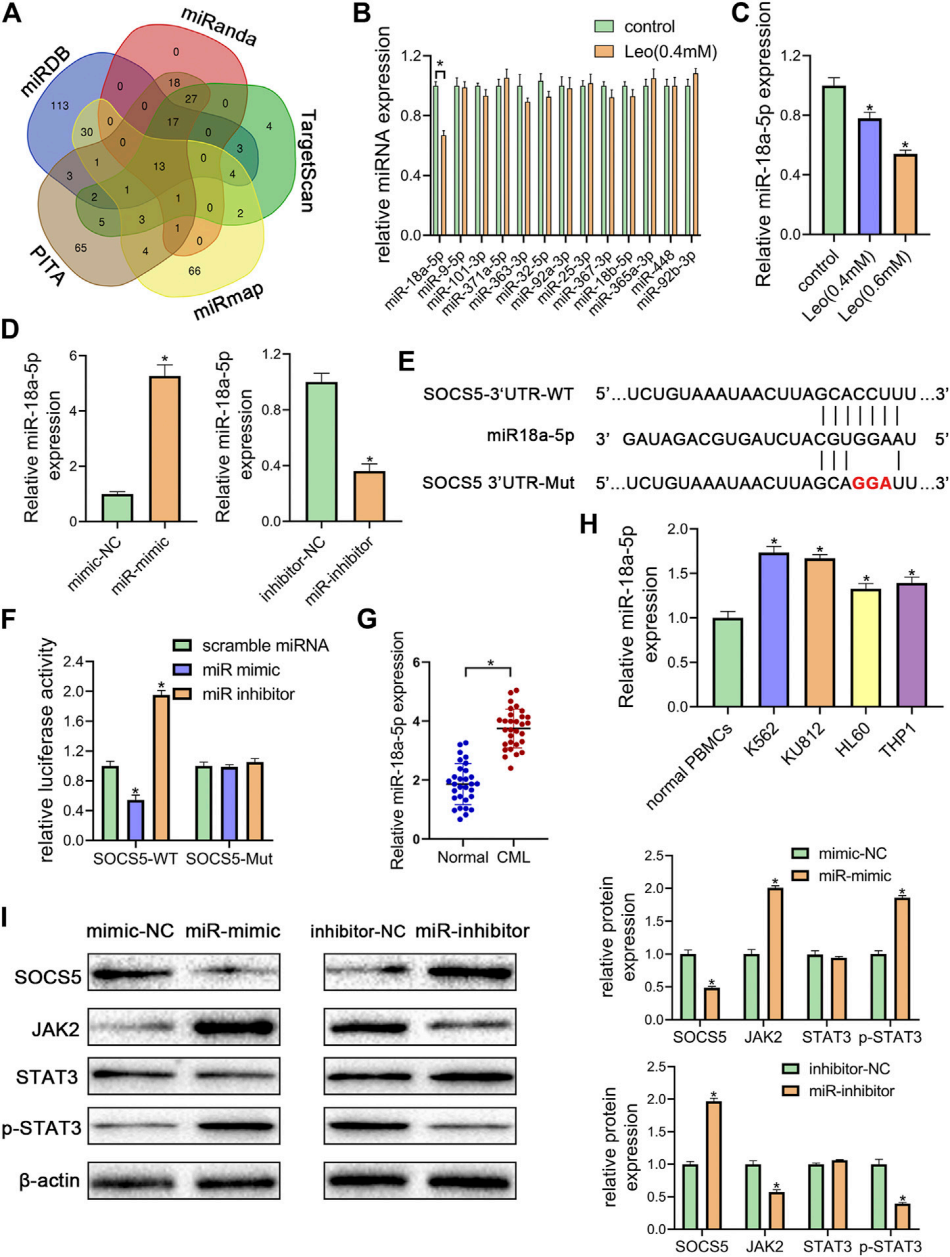
miR-18a-5p upregulation in CML cells represses SOCS5 expression through directly targeting its 3′-UTR **(A)** The target microRNAs of SOCS5 were predicted by five prediction algorithms. the Venn diagram showed an overlap of 13 microRNAs **(B)** K562 cells were treated with leonurine (0.4 mM) for 24 h qRT-PCR was used to detect the expressions of 13 potential miRNAs **(C)** K562 cells were treated with leonurine (0.4 and 0.6 mM) for 24 h qRT-PCR was used to detect the expression of miR-18a-5p **(D)** miR-18a-5p expression after miR-18a-5p mimic, mimic-NC, miR-18a-5p inhibitor, or inhibitor-NC transfection in K562 cells **(E)** The schematic diagram of wild 3′-UTR of SOCS5 with predicted miR-18a-5p target sites (SOCS5-WT) or the mutated sites of 3′UTR of SOCS5 (SOCS5-Mut) **(F)** Luciferase activity of K562 cells transfected with plasmids carrying a wild-type or mutant 3′UTR of SOCS5 in response to miR-18a-5p mimic or inhibitor **(G)** The expression of miR-18a-5p in PBMCs of CML patients and healthy donors **(H)** The expression of miR-18a-5p in leukemia cell lines and PBMCs of healthy donors **(I)** SOCS5 and JAK2/STAT3 expression was analyzed by western blotting. Results were presented as mean ± SD, and the error bars represent the SD of three independent experiments (*n* = 3); *p < 0.05 vs. control group.

To further illuminate the upstream mechanisms of SOCS5, the microRNAs targeting SOCS5 were predicted by five target prediction programs, miRanda, TargetScan, miRDB, PITA and miRmap. By selecting the intersection, we determined 13 potential miRNAs targeting 3′-UTR of SOCS5 (Figure 4A). The expression of these microRNAs was examined and only miR-18a-5p expression was dramatically reduced after leonurine treatment in K562 cells (Figures 4B,C). We transfected miR-18a-5p mimic or its inhibitor into K562 cells. The data exhibited that miR-18a-5p expression was dramatically increased in K562 cells transfected with miR-18a-5p mimic and decreased in cells transfected with its inhibitor (Figure 4D). Computer-based sequence analysis identified a complementary site of miR-18a-5p in the 3′-UTR of SOCS5 mRNA (Figure 4E). To verify that SOCS5 might be a downstream target of miR-18a-5p, we employed luciferase reporter assay. The results demonstrated that co-transfection of SOCS5-3′UTR wild-type and miR-18a-5p mimic resulted in greatly decreased luciferase activity relative to co-treatment with scrambled miRNA. This reduction, however, was rescued in K562 cells transfected with SOCS5-3′UTR Mut or miR-18a-5p inhibitor (Figure 4F).

Furthermore, we confirmed that the expression of miR-18a-5p was remarkably higher in CML PBMCs than in normal ones (Figure 4G). we also detected miR-18a-5p expression in different CML cell lines (K562 and KU812) and AML cell lines (HL-60 and THP-1). The data displayed that highest expression levels were observed in CML cells **(** Figure 4H).

To investigate the effect of miR-18a-5p on SOCS5/JAK2/STAT3, the protein expressions were further examined. As shown in Figure 4I, transfection of miR-18a-5p mimic dramatically reduced SOCS5 expression and increased JAK2 and p-STAT3 level, whereas silencing of miR-18a-5p by its inhibitor increased SOCS5 level and reduced JAK2 and p-STAT3 level. Similar results were observed in KU812 cell line (Supplementary Figure S3). These results manifested that miR-18a-5p upregulation-suppressing SOCS5 expression was closely related to CML pathological process.

### Leonurine Inhibits Growth and Enhances Apoptosis of CML Cells by Relieving miR-18a-5p Repression of SOCS5

**FIGURE 5:**
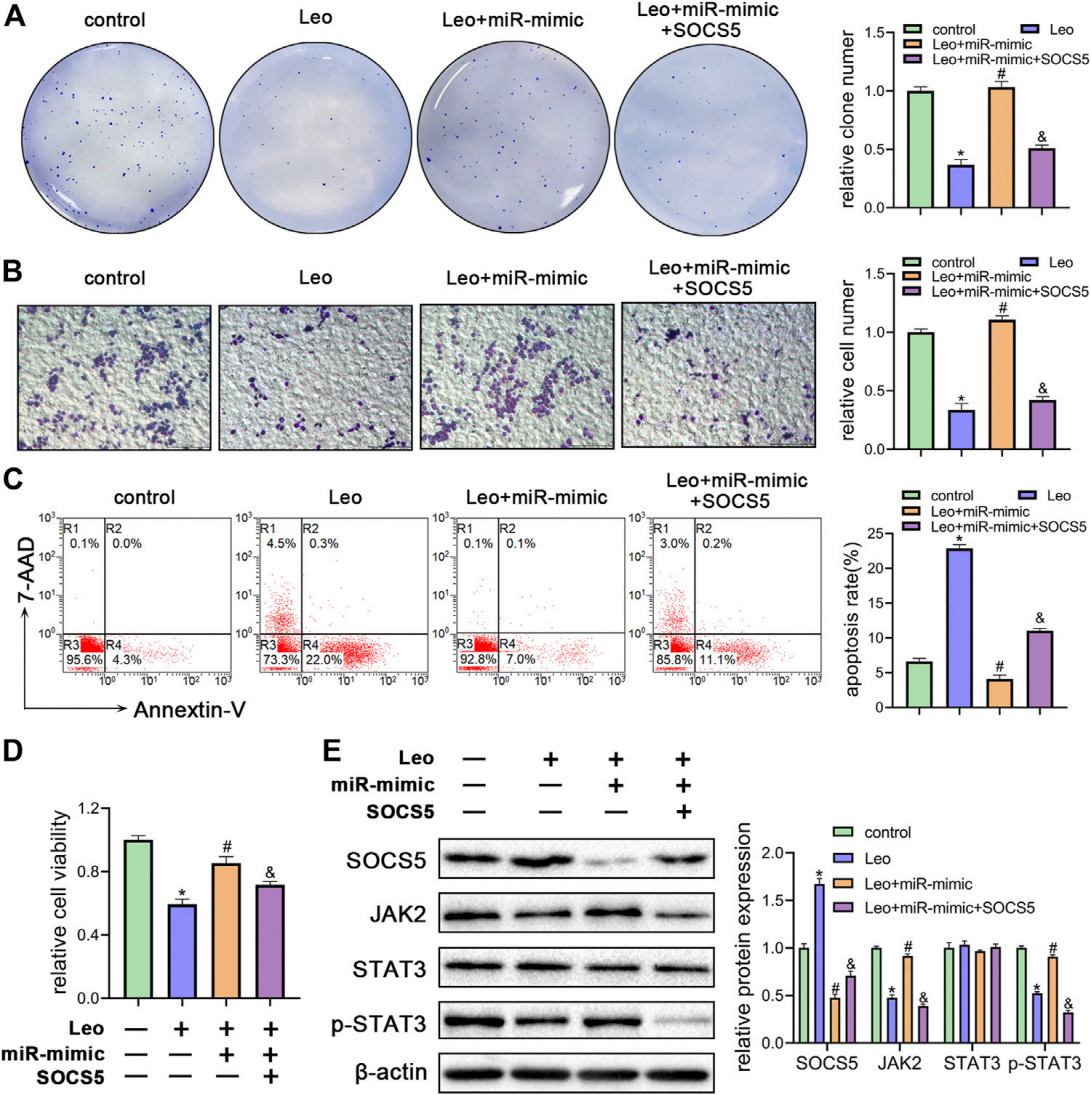
Leonurine inhibits growth and enhances apoptosis of CML cells by relieving miR-18a-5p repression of SOCS5 **(A)** K562 cells were treated with leonurine (0.4 mM) for 24 h alone or in combination with miR-18a-5p mimic and SOCS5. Colony formation ability was assessed by soft agar colony formation assay **(B)** Cell migration was analyzed by transwell assay. Scale bar = 100 μm **(C)** Cell apoptosis was assessed by Annexin V–FITC/7-AAD staining **(D)** Cell viability was detected by CCK-8 **(E)** Expression of SOCS5 and JAK2/STAT3 signaling was detected by western blotting. Results were presented as mean ± SD, and the error bars represent the SD of three independent experiments (*n* = 3); *p < 0.05 vs. control group; #*p* < 0.05 vs. Leo group; &*p* < 0.05 vs. Leo + miR-mimic group.

To further understand whether leonurine decreased cell growth and induced cell apoptosis by regulating miR-18a-5p/SOCS5 activity, K562 cells were treated with leonurine alone or in combination with miR-18a-5p mimic or SOCS5. The inhibitory effect of leonurine on cell colony formation was attenuated by co-treatment with miR-18a-5p mimic, and the effect of miR-18a-5p mimic was abolished by overexpression of SOCS5 (Figure 5A). Similar cell migration and cell viability results were also obtained in K562 cells under the same treatment (Figures 5B,D). On the contrary, leonurine treatment increased cell apoptosis, whereas miR-18a-5p mimic suppressed leonurine-induced apoptosis, and the effect of miR-18a-5p mimic was abrogated by SOCS5 overexpression (Figure 5C).

Next, we evaluated SOCS5/JAK2/STAT3 signaling activity by western blotting. As expected, miR-18a-5p mimic blocked leonurine-induced SOCS5 expression elevation, JAK2 and p-STAT3 expression decline, whereas SOCS5 overexpression reversed the effect of miR-18a-5p mimic (Figure 5E). Similar results were obtained in KU812 cell line (Supplementary Figure S4). These findings demonstrated that leonurine exerted anti-leukemia effect via regulating miR-18a-5p/SOCS5/JAK2/STAT3 axis.

### Leonurine Suppresses CML Xenograft Growth *In Vivo*

**FIGURE 6:**
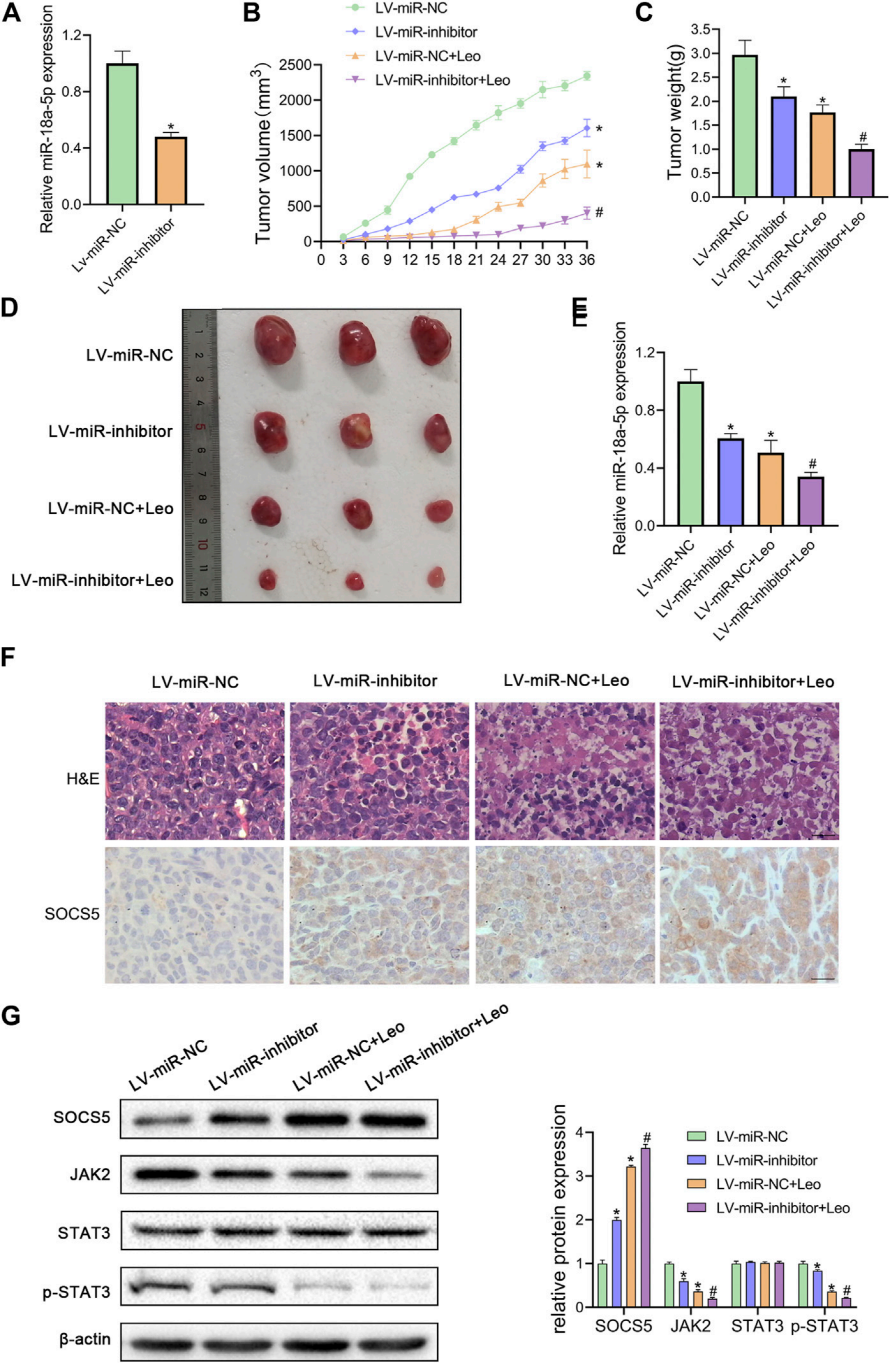
Leonurine suppresses CML xenograft growth *in vivo* **(A)** The expression of miR-18a-5p in K562 cells stably infected with lentivirus harboring miR-18a-5p inhibitor (LV-miR-inhibitor) or negative control (Lv-miR-NC) **(B)** Cells were injected subcutaneously into nude mice. From the eighth day, leonurine (150 mg/kg **D)** was administrated to mice intragastrically for 4 weeks. The tumor volume was assessed every 3 days. On day 36, mice were sacrificed to harvest tumors **(C)** Xenograft tumors were removed from mice and weighted **(D)** Image of tumors excised from mice **(E)** The expression of miR-18a-5p of excised tumors was detected by qRT-PCR **(F)** Tumors were sectioned for H&E stained and immunohistochemical. Scale bar = 20 μm **(G)** The protein expression of SOCS5 and JAK2/STAT3 signaling were determined by western blotting. Results were presented as mean ± SD. *p < 0.05 vs. control group; #*p* < 0.05 vs. LV-miR-inhibitor group; *n* = 3.

Next, we explored whether leonurine inhibits CML cell growth via regulating miR-18a-5p *in vivo*. First, K562 cells stable suppression of miR-18a-5p (LV-miR-inhibitor) and the control cells (LV-miR-NC) were established using lentiviral vectors. As illustrated in Figure 6A, LV-miR-inhibitor downregulated miR-18a-5p expression. Subsequently, nude mice were inoculated subcutaneously with established cells and then treated with or without leonurine. The tumor volume and weight were observed. Our results revealed that leonurine or miR-18a-5p inhibition alone dramatically decreased tumor growth. Moreover, leonurine treatment together with miR-18a-5p inhibition further reduced the volume and weight of tumors (Figures 6B–D). We also found leonurine significantly inhibited miR-18a-5p expression in xenograft tumors (Figure 6E). H&E staining and immunohistochemical showed that leonurine together with miR-18a-5p suppression significantly destroyed the morphology of tumor tissues and increased the expression of SOCS5 protein (Figure 6F). We further detected the expression of SOCS5/JAK2/STAT3 signaling in xenograft tumors by western blotting. The results showed that SOCS5 level was significantly upregulated in leonurine-treated or miR-18a-5p–suppressed mice, especially in their combination. And the expression of JAK2 and p-STAT3 was obviously reduced in leonurine-treated or miR-18a-5p–suppressed mice (Figure 6G). These results indicated that leonurine suppressed CML cell growth *in vivo* by modulating miR-18a-5p/SOCS5/JAK2/STAT3 axis.

## Discussion

In this study, we investigated the potential benefits of leonurine for CML treatment and underlying mechanisms. Our results indicated that leonurine inhibited proliferation, colony formation, migration and induced apoptosis of CML cells. These effects were associated with upregulation of SOCS5. Mechanistically, we revealed that miR-18a-5p directly targets SOCS5 to boost JAK2/STAT3 signaling activity. Furthermore, SOCS5 expression was lower and miR-18a-5p expression was higher in CML patients than normal ones. *In vivo* experiments verified that leonurine inhibited CML xenograft growth by repressing miR-18a-5p/SOCS5/JAK2/STAT3, suggesting a potential approach to CML therapy.

JAK2 leads to the recruitment and activation of STAT3 to promote tumorigenesis by accelerating proliferation and inhibiting apoptosis. The dysregulation of JAK2/STAT3 pathway plays a critical role in CML pathogenesis (Sattler and Griffin, 2003; Cheng et al., 2018). To explore whether JAK2/STAT3 pathway is involved in leonurine-mediated CML cell response, JAK2 and STAT3 protein expressions were detected. We found leonurine reduced JAK2 and phosphorylated STAT3 level. SOCS5, a member of the SOCS family, inhibits the activity of JAK tyrosine kinase. A lot of evidences suggest that SOCS5 serves as a tumor suppressor in cancers by negatively regulating JAK2/STAT3 (Zhang Z. et al., 2019; Yang et al., 2020). Although SOCS5 dysregulation has been recently associated with acute and chronic lymphocytic leukemia (Toniolo et al., 2016; Sharma et al., 2019), its role in CML has not been characterized to date. In the present study, we confirmed that SOCS5 was downregulated in CML cell lines and PBMCs from CML patients. Leonurine treatment significantly increased SOCS5 expression and decreased the expression of JAK2/STAT3. Moreover, the effect of leonurine was impaired by SOCS5 knockdown. Our results indicated that SOCS5/JAK2/STAT3 was involved in anti-leukemia process of leonurine and that SOCS5 may be a potential diagnostic marker and therapeutic target for CML. We noted leonurine did not completely inhibit the expressions of JAK2 and p-STAT3. It has been reported previously that leonurine served an antitumor role through various mechanisms. For example, leonurine inhibits lung cancer cells by a mitochondria-dependent pathway and suppresses glioma cells by regulating DNA damage and epigenetic modification (Mao et al., 2015; Sitarek et al., 2017). For our study, we concluded leonurine exerted its anti-leukemia effect at least partially by suppressing JAK2/STAT3 signaling pathway. There may exist other signaling pathways that, together with JAK2/STAT3, are involved in the anticancer effects of leonurine. This, indeed, requires further studies. In addition, we demonstrated that leonurine could improve the vulnerability of CML cells to imatinib by suppressing proliferation and inducing apoptosis. Moreover, by analyzing a published microarray data, we found SOCS5 was significantly upregulated after imatinib treatment in CML patients. As SOCS5 is upregulated by both leonurine and imatinib treatment, the mechanism by which leonurine enhances the effect of imatinib deserves further evaluation in our future study.

miRNAs have been shown to play a crucial role by modulating gene expression in tumor development and progression. SOCS5 expression is regulated by several miRNAs. miR-522–3p was found to directly target SOCS5 to promote lung cancer cell viability, migration and invasion (Fan et al., 2020). A recent study showed that SOCS5 was a direct target of miR-675–3p, which activated PI3K signaling to maintain pancreatic cancer cell stemness (Wang et al., 2020). SOCS5 was also identified as the target of miR-9-5p in cervical cancer cells (Wei et al., 2019). Notably, a previous study showed that miR-18a-5p could target SOCS5 and regulate its expression in cholangiocarcinoma (Peng et al., 2020). In liver cancer, upregulation of miR-18a and miR-25 inhibited the expression of SOCS5, which was identified as a *bona fide* target of both miRNAs (Sanchez-Mejias et al., 2019). miR-18a belonging to the miR-17–92 cluster has been found to either promote or inhibit tumorigenesis, suggesting its dual function in cancer progression. On the one hand, miR-18a-5p was demonstrated to play an oncogenic role by regulating downstream targets and promoting cancer progression in various cancer types such as lung cancer (Liang et al., 2017), cervical cancer (Morgan et al., 2020), prostate cancer (Hsu et al., 2014) and gastric cancer (Wu et al., 2013; Tsujiura et al., 2015). On the other hand, miR-18a-5p was found to inhibit malignant progression of breast cancer (Godfrey et al., 2013; Zhang N. et al., 2019), colorectal cancer (Vega et al., 2013) and pancreatic cancer (Li et al., 2017). These studies suggest the dual function of miR-18a-5p in cancer progression. As for hematological malignancies, miR-18a-5p was found to be significantly overexpressed in AML cells (Zhang et al., 2020). Neda et al. found lower expression of miR-18a-5p in pediatric patients with acute lymphoblastic leukemia (ALL) than in adult ones, which may explain the better prognosis of pediatric ALL (Mosakhani et al., 2017). A study assessed expression profiles of several oncogenic miRNAs and showed that miR-18a was elevated in imatinib-resistant compared to imatinib-responsive patients (Jurkovicova et al., 2015). Here, our study indicated that miR-18a-5p was significantly upregulated and miR-18a-5p repressed SOCS5 expression by directly targeting its 3′-UTR in CML cells. Overexpression of miR-18a-5p could avoid the growth inhibition effect of leonurine. Nonetheless, enforced expression of SOCS5 relieved miR-18a-5p from inhibiting the effect of leonurine, which indicates leonurine downregulates miR-18a-5p to upregulate SOCS5 expression and thus attenuates JAK2/STAT3 pathway activity. More importantly, we further verified leonurine exerted anti-leukemia effect via downregulating miR-18a-5p *in vivo*. As a miRNA has more than one target genes (Ambros, 2004), other target genes of miR-18a-5p may also exist and play roles in CML. Therefore, further research is needed to fully understand the role of miR-18a-5p in CML development.

Studies have reported that leonurine exhibited anticancer effect in various solid tumor cells. In present study, we demonstrate for the first time that leonurine has an anti-leukemia effect. Our data confirmed that leonurine suppressed the biological activity of CML cells both *in vitro* and *in vivo*. What is more, leonurine had little inhibitory effect on normal PBMCs even at high concentrations, revealing it has a very low toxicity and is well tolerated. As leonurine was proven to exert protective effect against oxidative stress and inflammation at low concentrations (Liu et al., 2010), we suspect that leonurine might have protective effect on normal cells and thus could reduce side effects of other drugs, without affecting their effectiveness. Besides, we verified leonurine could enhance the sensitivity of CML cells to imatinib, indicating leonurine as a promising adjuvant for CML treatment. In the future, we will focus on the combined effect of leonurine with other drugs.

## Conclusion

In summary, leonurine inhibits proliferation, migration and induces apoptosis of CML cells via regulating miR-18a-5p/SOCS5/JAK2/STAT3 signaling. Therefore, leonurine may provide a new and safe therapeutic approach for CML treatment in future.

## Data Availability

The original contributions presented in the study are included in the article/Supplementary Material, further inquiries can be directed to the corresponding authors.
