# Supplementary figures and images for: Leonurine-Repressed miR-18a-5p/SOCS5/JAK2/STAT3 Axis Activity Disrupts CML malignancy
Source: Front Pharmacol. 2021 Apr 16;12:657724. doi: 10.3389/fphar.2021.657724 (PMC8087248; doi:10.3389/fphar.2021.657724)

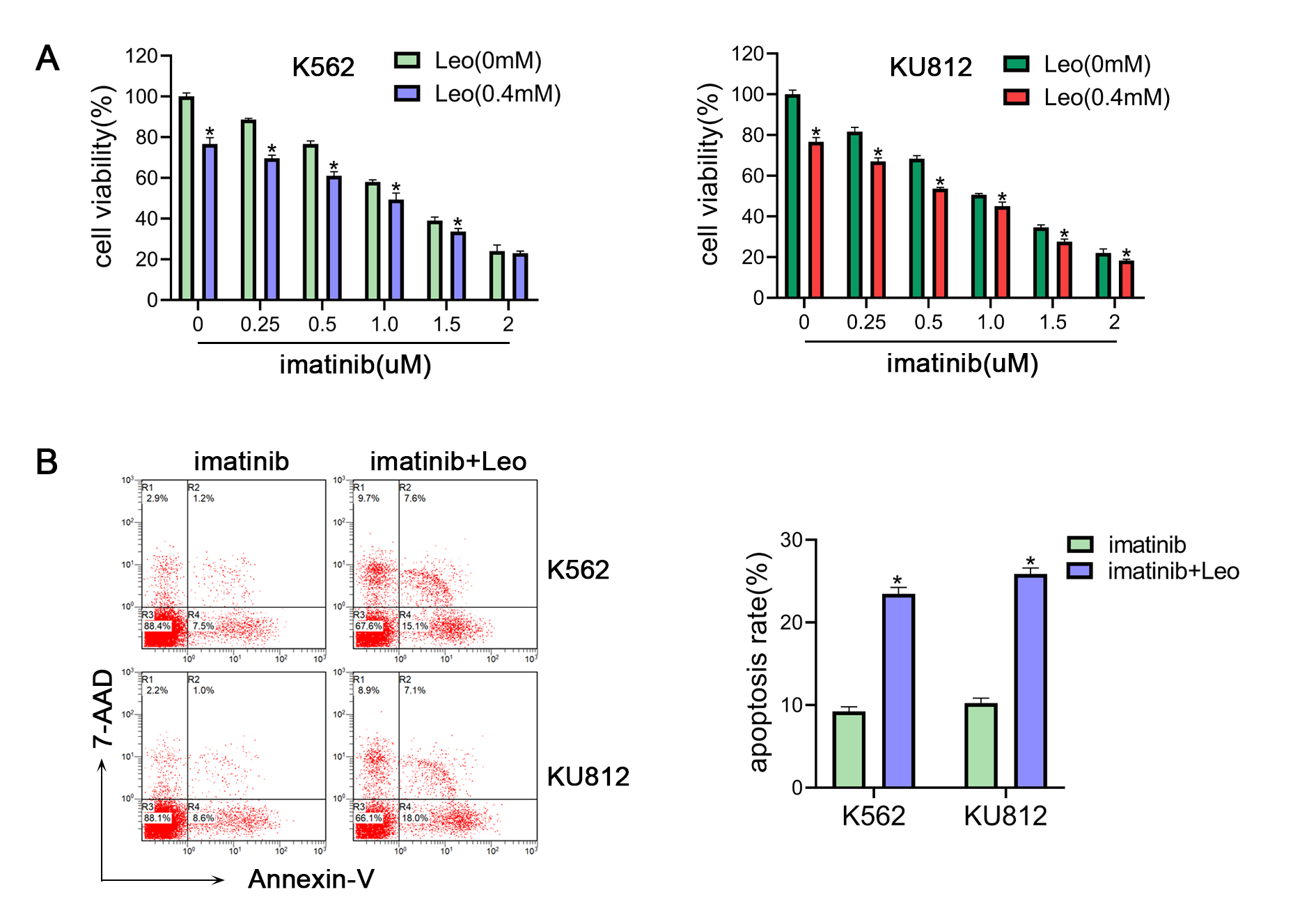

Supplement: Supplementary file 1 [file image1.tif]

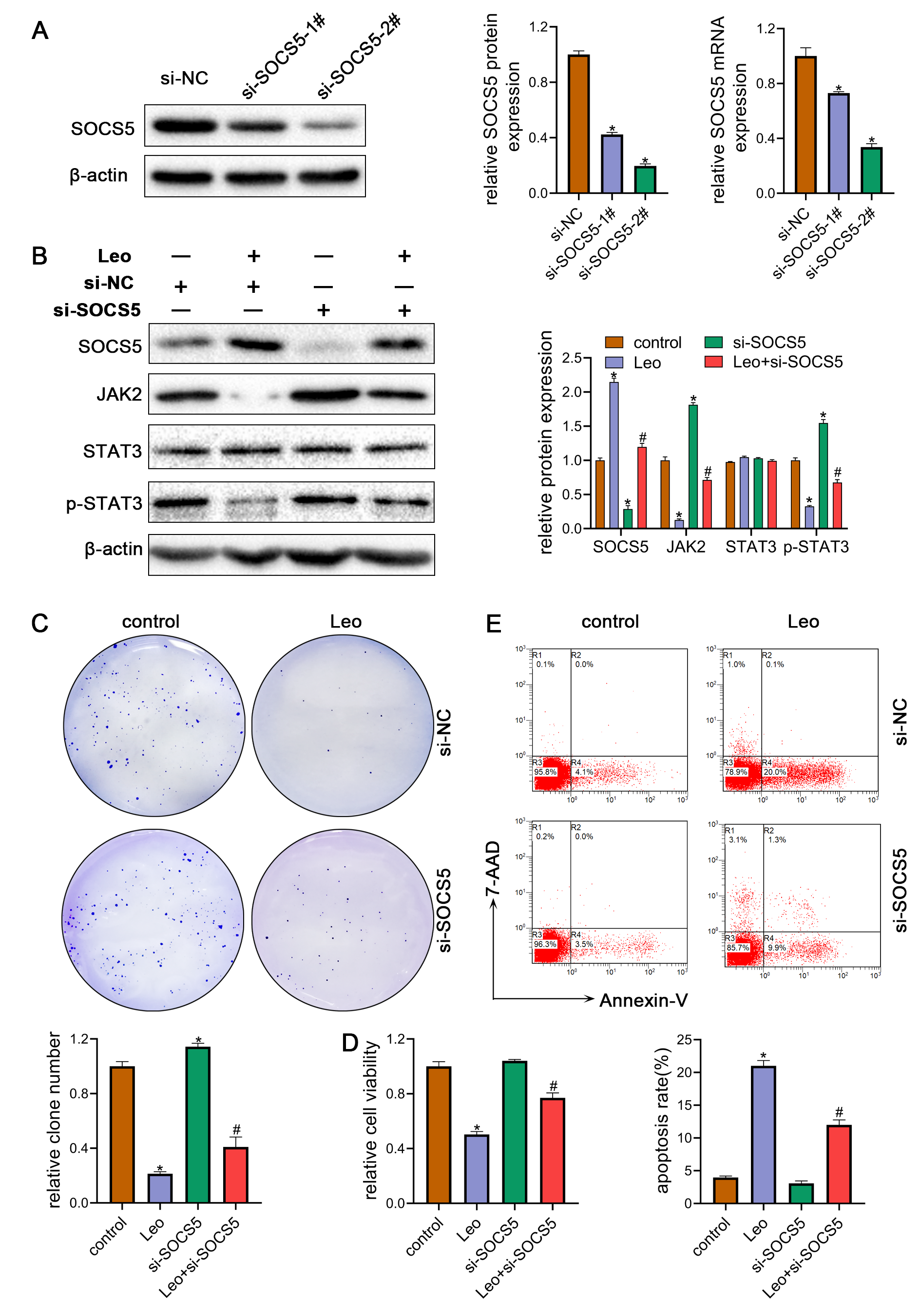

Supplement: Supplementary file 2 [file image2.tif]

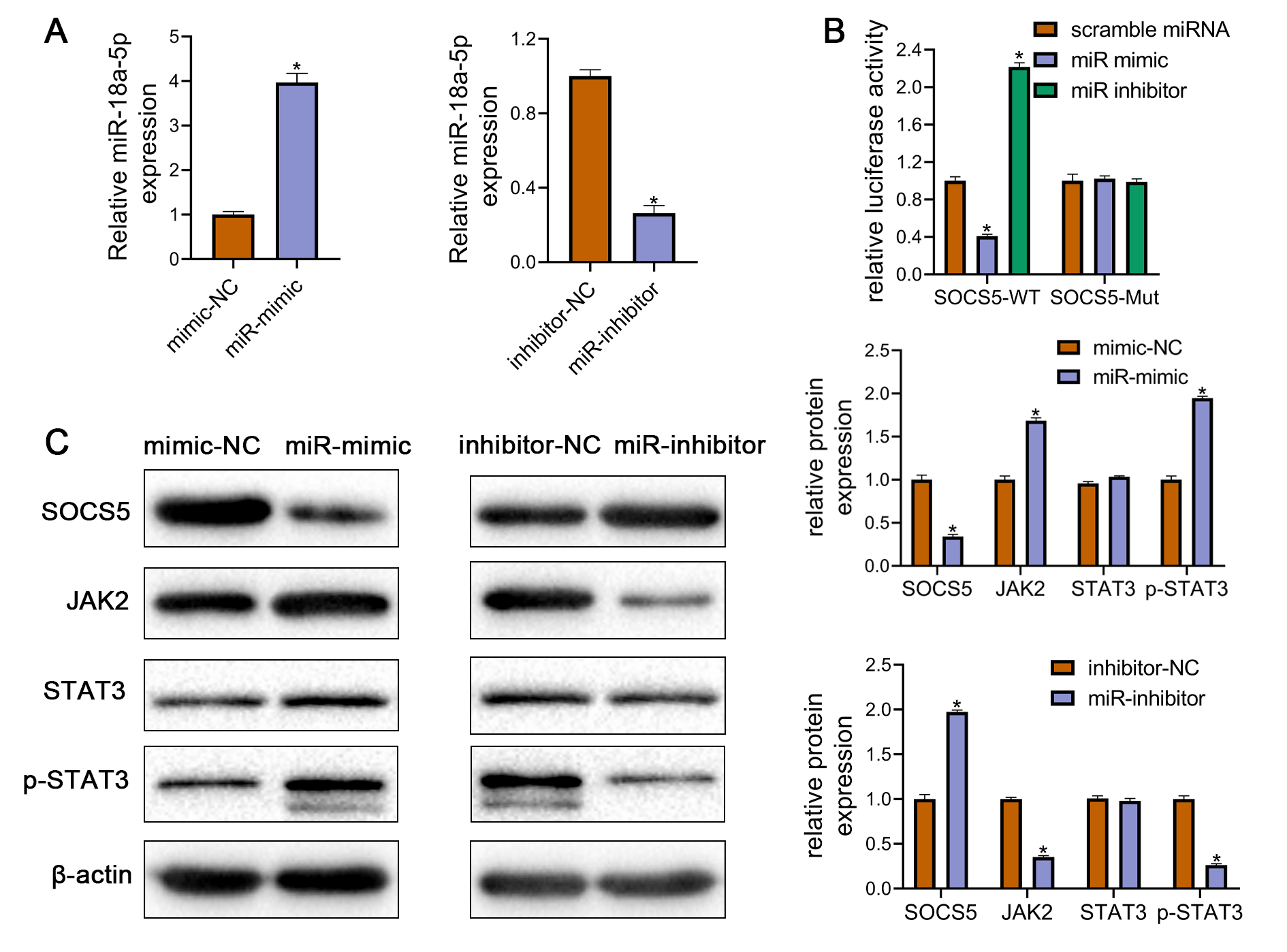

Supplement: Supplementary file 3 [file image3.tif]

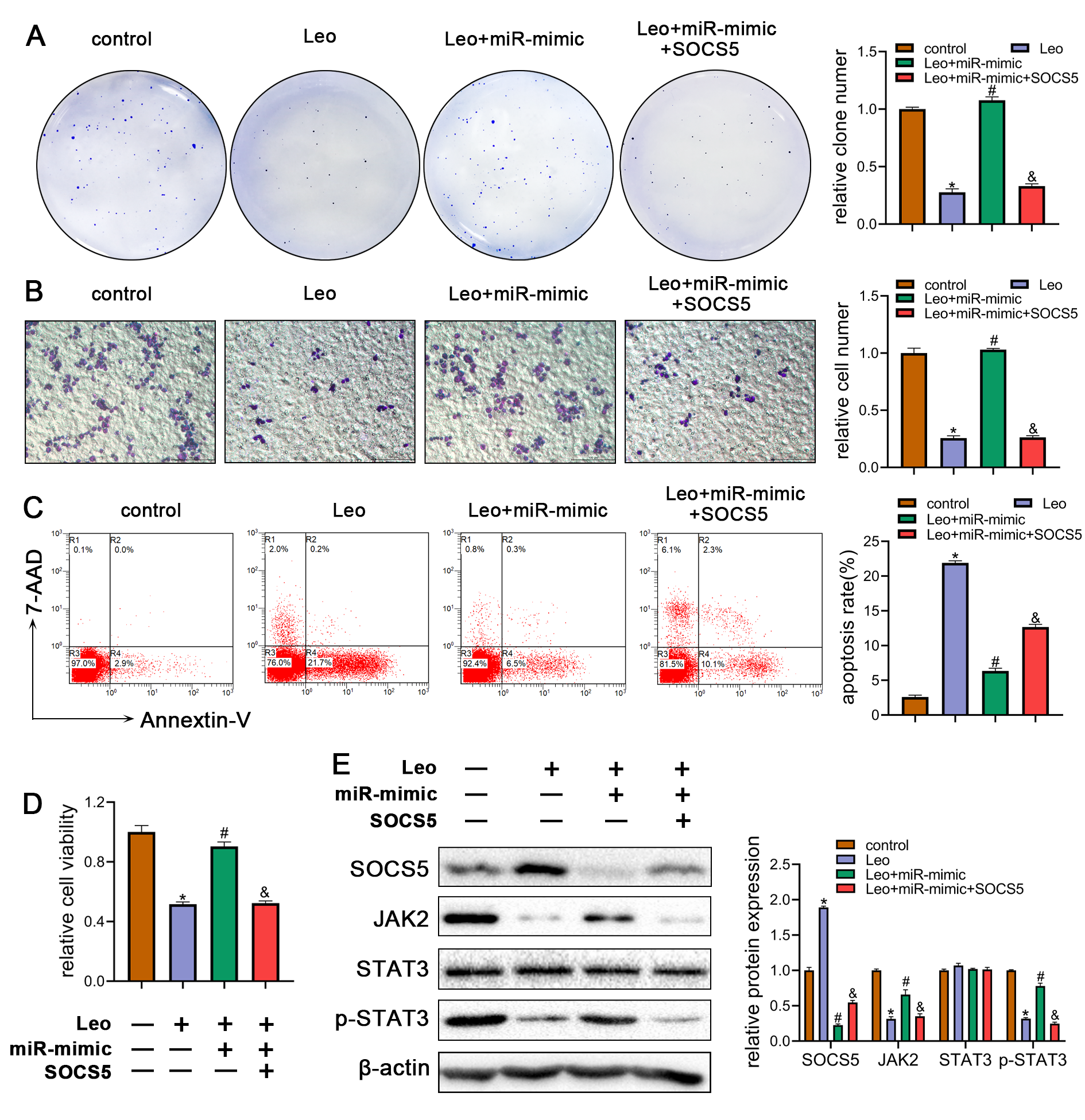

Supplement: Supplementary file 4 [file image4.tif]
